# Hereditary spastic paraplegia (SPG 48) with deafness and azoospermia: A case report

**DOI:** 10.3389/fneur.2023.1156100

**Published:** 2023-04-03

**Authors:** Ping Jin, Yu Wang, Na Nian, Gong-Qiang Wang, Xiao-Ming Fu

**Affiliations:** ^1^Department of Neurology, The Affiliated Hospital of Institute of Neurology, Anhui University of Chinese Medicine, Hefei, China; ^2^Institute of Neurology, Anhui University of Chinese, Hefei, China

**Keywords:** *AP5Z1* gene mutation, SPG48, azoospermia, impaired hearing, brain MRI

## Abstract

Hereditary spastic paraplegias (HSP) are inherited neurodegenerative disorders characterized by progressive paraplegia and spasticity in the lower limbs. SPG48 represents a rare genotype characterized by mutations in *AP5Z1*, a gene playing a role in intracellular membrane trafficking. This study describes a case of a 53-year-old male patient with SPG48 presenting spastic paraplegia, infertility, hearing impairment, cognitive abnormalities and peripheral neuropathy. The Sanger sequencing revealed a homozygous deletion in the chr 7:4785904-4786677 region causing a premature stop codon in exon 10. The patient's brother was heterozygous for the mutation. The brain magnetic resonance imaging found a mild brain atrophy and white matter lesions. In the analysis of the auditory thresholds, we found a significant hearing decrease in both ears.

## Introduction

Hereditary spastic paraplegia (HSP) represents a rare group of neurodegenerative disorders characterized by progressive lower limb spasticity and weakness (1). HSP can be inherited in an autosomal dominant, autosomal recessive, X-linked recessive or mitochondrial manner (2). Several genotypes (from SPG1 to SPG82) and pathogenic genes have been identified, with the most common causative genes being *SPAST, ATL1* and *REEP1* (3). Damaging variants in *AP5Z1* are associated with SPG48, a rare autosomal recessive condition with a variegated clinical phenotype (4). To date, we know of 14 case reports related to this gene. In this study, we described the clinical features and imaging findings of a 43-year-old Chinese male patient with a large deletion in the *AP5Z1* gene. The patient reported spastic paraplegia, hearing impairment and infertility.

## Case presentation

During April 2020, a 53-year-old Chinese male patient was admitted to our hospital due to progressive walking difficulties and distal weakness at lower limbs. He got low grades during primary school, and then did not complete it. He married at the age of 28 years. When he was 35 years old, he received a diagnosis of infertility. In 2010, the patient experienced instability and mild shaking during walking. Walking instability worsened in 2015, when the patient reported difficulties with standing up from a squatting position. In 2019, the patient started using crutches to walk. Since 2017, hearing capacity has gradually decreased in both ears. Since 2019, the patient only recognizes sounds spoken aloud, without pain, infection and/or tinnitus in the ears. The patient's parents were first cousins, the family history was unremarkable for similar neurologic and auditory findings.

**Figure 1:**
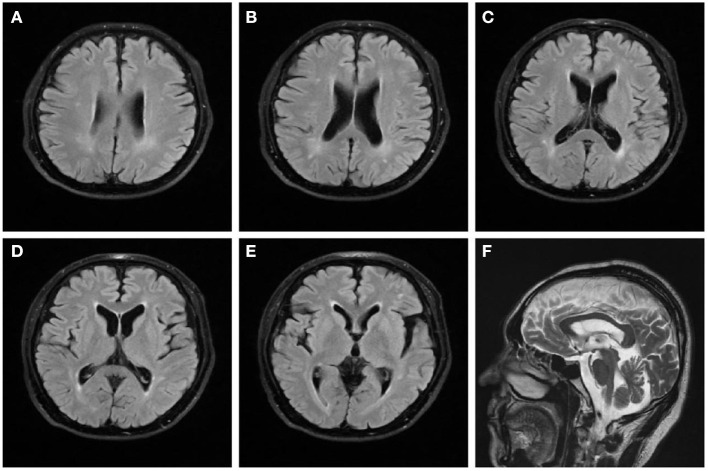
Brain magnetic resonance imaging of the patient. FLAIR sequences **(A–E)** reveal high signal changes in the periventricular white matter and mild brain atrophy. T2-weighted **(F)** sequence shows that the corpus callosum is normal.

On examination, we observed bilateral spastic gait, tendon hyperreflexia, ankle clonuses (+) and bilateral pyramidal signs. The Romberg's test was positive. A mild spastic dysarthria was also evident. Sensory examination revealed loss of vibratory sensations at the great toes and reduced pinprick sensations in to the lower limbs. The spastic paraplegia rating scale (SPRS) scored 19 points. The orientation in space and time was normal but attention and calculation wreere impaired. Hearing loss affected both ears, with positive left (+) and right (±) Rinne tests. The Weber test was biased toward the right ear. Both appearance and size of the testis were normal. Blood laboratory tests and cerebrospinal fluid evaluation were normal, including systemic hormones. Brain magnetic resonance imaging (MRI) showed a mild brain atrophy with periventricular white matter hyperintensities on fluid-attenuated inversion recovery (FLAIR) sequences (Figure 1). Cervical and lumbar MRI described degenerative changes in the lumbar spine and mild distension of multiple intervertebral disks, with no significant compression or other abnormal signals of the spinal cord. The electromyography investigation showed decreased amplitudes of compound muscle action potentials (CMAPs) in the right common peroneal nerve and left tibial nerve. A prolonged latency of sensory nerve action potentials (SNAPs) was observed in bilateral sural nerves, with an increased latency of the F wave in median and tibial nerves of both limbs. Bilateral tibial H-reflex investigation showed a prolonged latency, with a neurogenic damage in the tibial anterior muscles. The audiometry evaluation found a sensorineural deafness on the left side and a mixed deafness on the right side. The evoked potentials suggested an increased binaural auditory threshold. On the brain stem auditory evoked potentials, we found bilateral prolonged waves I and III. A prolonged left P40 latency was also described on somatosensory evoked cortical potentials. The results obtained on cognitive tests were 27 points on the Mini Mental State Examination (MMSE) and 18 points on the Montreal Cognitive Assessment (MoCA).

**Figure 2:**
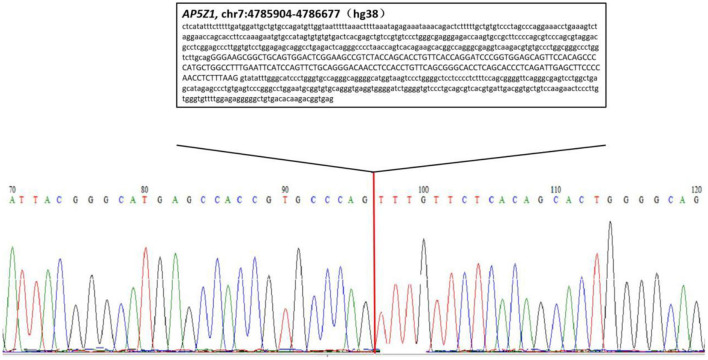
Sequencing chromatogram of the patient. The lower case letters represent the nucleotide sequence of intron regions while capital letters represent the nucleotide sequence of exon 10.

**Figure 3:**
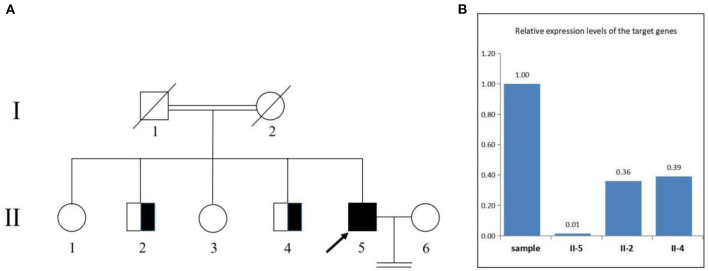
Pedigree of the family **(A)**. The deletion involving exon 10 of the AP5Z1 gene was verified by fluorescence quantitative polymerase chain reaction **(B)**. The squares indicate males, the circles indicate females. The pedigree chart shows that the patient's father (I-1) and mother (I-2) were cousins and deceased. The proband (II-5) is indicated by a black arrow. The patient's first brother (II-2) and second brother (II-4) were characterized by heterozygous mutations. The patient's sister refused to be tested.

We confirmed the location of gene deletion by Sanger sequencing, identifying homozygous deletions in the chr7:4785904-4786677 region involving exon 10 of the *AP5Z1* gene, NM_014855.3, c.1133-345_1311 + 249del, p.G378Vfs^*^93X (Figure 2). Using the software Mutalyzer 2.0.35, we found that the deletion causes early termination of amino acid synthesis. The patient's brothers were asymptomatic, with heterozygous mutations identified by fluorescent quantitative polymerase chain reaction (PCR). The primers were as follows: *AP5Z1*-10 (forward: AACCAGTCACAGAAGCACGG, reverse: GAACAGGTGGAGGTTGTCCC), and the sequences of the PCR products were determined using the ABI 9700 Genetic analyzer (ABI, Foster City, CA). Reaction system: total volume 10ul, including 1ul of genomic DNA template, 5ul of SYBR Green Mix, 0.5ul of upstream and downstream primers, 3ul of ddH_2_O. Reaction conditions: 95°C, 30s, (95°C, 15s; 60°C, 15s; 72°C, 15s) × 46 cycles. The final data was analyzed by 2^−ΔCt^. The results showed that relative to two copies of healthy people, the patient had 0 copies, his brothers had a single copy (Figure 3). This deletion mutation is considered a pathogenic variant according to American College of Medical Genetics and Genomics standards and guidelines. Based on clinical features, imaging findings and genetic abnormalities, we diagnosed the patient with SPG48. Oral administration of baclofen and tizanidine improved his symptoms.

## Discussion

In 2010, Slabicki et al. (5) identified SPG48 as a novel genotype associated with HSP (5). SPG48 can be defined as a secondary lysosomal storage disease involving adaptor proteins (APs), ubiquitously expressed protein complexes ranging from AP1 to AP5 (6). AP5 is a cation-independent mannose 6-phosphate receptor located in endosomes and lysosomes that transports Golgi membrane protein 1 to the Golgi apparatus (7). AP5 is expressed in non-neuronal and neuronal tissues, such as cerebral cortex, hippocampus and cerebellum. Neuronal cells are abnormally sensitive to dysfunction of lysosomes and protein accumulation (8). The subtype SPG48 is associated with mutations in the zeta subunit of AP5, impairing the complex formation of AP5. This, in turn, results in defects in lysosomal structure and function (9). A mouse model of SPG48 showed that loss-of-function AP5 variants blocked autophagy, leading to an aberrant accumulation of autophagic vacuoles and axon degeneration (10). Postmortem findings in patients with bi-allelic *AP5Z1* mutations demonstrated an extensive degeneration of cortical and subcortical regions, including basal ganglia, brainstem and spinal cord (8). Such alterations are common in neurodegenerative disorders, including HSP, amyotrophic lateral sclerosis and spinocerebellar ataxia.

As of today, 14 cases of SPG48 have been reported worldwide (Table 1). Most of them were characterized by point mutations, whereas our patient reported a segmental deletion in the *AP5Z1* gene. Overall, the age of onset of SPG48 spans many decades and clinical manifestations are highly heterogeneous, including spastic paraplegia, urinary incontinence, ataxia, intellectual disability, sensorimotor neuropathy, Parkinson's syndrome, dystonia and eye disturbances [including pigmentary retinopathy, optic atrophy, cataract, glaucoma and ophthalmoplegia (11)], the atypical SPG48 did not even show spastic paraplegia (8). In most patients, brain MRI shows white matter lesions around corona radiata, semioval center and lateral ventricles. The “ears of the lynx” imaging sign suggests the presence of a genetic mutation, likely characteristic of HSP (12). The narrowing of corpus callosum is another relevant imaging characteristic in these patients. A few patients showed a normal brain MRI, whereas individual cases reported a diffuse brain atrophy and/or abnormal spinal cord signals.

**Table 1:** Clinical, radiological and genetic features of SPG48/*AP5Z1* patients.

**References**	**Patient no**.	**Sex/age at onset**	**Nationality**	**Genotype**	**Clinical symptoms**	**MRI**
Słabicki et al. (5)	1	F/50y	French	c.80_83del4, homozygous	Spastic paraplegia, urinary incontinence	Normal
Słabicki et al. (5)	2	M/49y	French	79_84ins22(p.R27Lfs^*^3), homozygous	Spastic paraplegia, urinary incontinence	Spinal hyperintensities at C3–C4 and C7
Pensato et al. (17)	3	F/47y	Italian	c.412C>T(p.R138^*^) and c.1322G>A(p.W441^*^), compound heterozygous	Spastic paraplegia, urinary incontinence	Severe narrowing of corpus callosum and white matter hyperintensity at the frontal horns of lateral ventricles
Pensato et al. (17)	4	F/2y	Moroccan	c.616C>T(p.R206W), homozygous	Spastic paraplegia, mild intellectual disability	Mild narrowing of corpus callosum with periventricular white matter hyperintensities
Schlipf et al. (1)	5	F/43y	German	c.874C>T(p.R292W) and c.2267C>T(p.T756I), heterozygous	Cerebellar dysfunction, myokymia, bilateral congenital nystagmus	Normal
Hirst et al. (8)	6	M/60y	German	c.1732C>T(p.Q578^*^), homozygous	Spastic paraplegia, spastic bladder, spastic dysarthria, parkinsonism, limb ataxia, mild motor and sensory polyneuropathy, pigmentary retinopathy, cataracts, macular thinning, foot dystonia, mild hearing loss	Diffuse atrophy and “ears of the lynx” sign
Hirst et al. (8)	7	M/39y	Belgian	c.412C>T(p.R138^*^) and c.1033C>T(p.R345^*^), heterozygous	Spastic paraplegia, spastic bladder, parkinsonism, limb ataxia, moderate axonal mixed polyneuropathy/ distal amyotrophy, pigmentary retinopathy, cataracts, mild intellectual disability, spastic ataxic gait	White matter lesions
Hirst et al. (8)	8	F/40y	Belgian	c.412C>T(p.R138^*^) and c.1033C>T(p.R345^*^), heterozygous	Spastic paraplegia, spastic bladder, parkinsonism, limb ataxia, dysarthria, glaucoma, bilateral pigmentary retinopathy, mild cataracts, lens sclerosis, hypometric saccades, moderate axonal mixed polyneuropathy, mild intellectual disability, limb dystonia, spastic ataxic gait	White matter lesions
Hirst et al. (8)	9	M/52y	Belgian	c.412C>T(p.R138^*^) and c.1033C>T(p.R345^*^), heterozygous	Spastic paraplegia, spastic bladder, limb ataxia, pigmentary retinopathy, cataracts, slow saccades, spastic ataxic gait, mild intellectual disability, distal	White matter lesions
Hirst et al. (8)	10	F/Childhood		c.1364C>T(p.P455L), homozygous	sensory-motor polyneuropathy, amyotrophy	Normal
Hirst et al. (8)	11	M/13y	Kuwaitis	c.500C>A(p.T167N) and c.2010C>A(p.F670L), heterozygous	Spastic paraplegia, spastic bladder, limb ataxia, hypometric saccades, mild intellectual disability, intellectual regression at age 13, myoclonus, limb dystonia	White matter lesions and thinning of corpus callosum
D'Amore et al. (18)	12	NA	Italian	c.1302-1G>T and c.2287G>A(p.V763M), heterozygous	NA	NA
Wei et al. (4)	13	M/58y	Chinese	c.164C>T(p.T55M) and c.923G>C(p.S308T), heterozygous	Spastic paraplegia, sensory and cerebellar signs were absent, peripheral neuropathy	Normal
Maruta et al. (3)	14	F/47y	Japanese	c.1662_1672del(p.Q554Hfs^*^15), homozygous	Spastic paraplegia, cramps in foot and hands	White matter lesions and narrowing of corpus callosum
Our case	15	M/53y	Chinese	c.1133-345_1311+249del (p.G378Vfs^*^93X), homozygous	Spastic paraplegia, severe bilateral hearing loss, azoospermia, mild cognitive impairment, peripheral neuropathy	White matter lesions and mild brain atrophy

F, female; M, male; MRI, magnetic resonance imaging; NA, not described in the studies. The ^*^ symbol indicates the stop codon.

Our patient reported spastic paraplegia, peripheral neuropathy and mild cognitive impairment, and for the first time findings such as azoospermia and severe bilateral hearing loss. Previous studies found a significant reduction in mitochondrial length and density in HSP neurons, suggesting that an abnormal mitochondrial morphology may contribute to axonal defects (13). A recent study found that an impaired mitochondrial dynamics contributed to axonal degeneration in SPG11 and SPG48 neurons, resulting in less efficient and shortened axons (14). Mitochondrial function is also related to human sperm motility and morphology (15). In addition, loss of mitochondrial function has been associated with deafness (16). Therefore, we might speculate that *AP5Z1* gene mutations may affect both spermatogenesis and hearing.

SPG48 greatly impacts the central nervous system, with complex and varying clinical and radiographic characteristics. Clinicians should be aware of non-neurological findings in the presence of suspected SGP48 cases, as early recognition of the disease will minimize unnecessary evaluations and treatments. At present, there is no effective treatment for SPG48, symptomatic and supportive treatment including baclofen and tizanidine can moderately improve the symptoms of spastic paraplegia and urinary incontinence. Future therapies might restore mitochondrial function.

## Data availability statement

The original contributions presented in the study are included in the article/supplementary material, further inquiries can be directed to the corresponding author.

## Ethics statement

Written informed consent was obtained from the individual(s) and/or minor(s)' legal guardian/next of kin for the publication of any potentially identifiable images or data included in this article.

## Author contributions

PJ wrote the manuscript with input from all other authors. All authors contributed to the article and approved the submitted version.
